# Supplementary material for: Patterns of Intron Gain and Loss in Fungi
Source: PLoS Biol. 2004 Nov 30;2(12):e422. doi: 10.1371/journal.pbio.0020422 (PMC532390; doi:10.1371/journal.pbio.0020422)
Supplement: Table S1 — Also available at http://genes.mit.edu/NielsenEtAl/. (4.3 MB ZIP). [file pbio.0020422.st001.zip › NielsenEtAl/html/225.html]

AN4483.1.NCU09212.1.MG08547.1.FG00786.1


```
 CLUSTAL W (1.82) Multiple Sequence Alignments - Introns Inserted


Sequence 1: NCU09212.1	612 aa
Sequence 2: MG08547.1	580 aa
Sequence 3: FG00786.1	574 aa
Sequence 4: AN4483.1	686 aa
Alignment Length: 728 aa
Number Identitical Residues: 306 aa
Alignment Score (without introns) 14106


MG08547.1 	--------------~-------------------------------MAPVSEPAFGGVP-
NCU09212.1	MSTIQQLKNFIRHG1KQARTANPEEPARTKQADHSATQHHHQPTKMAAPVSEPNLGGSPP
FG00786.1 	MSTIQQLKNFIRHG1KQAR---------------------------AAPEEAP-------
AN4483.1  	MSTIQSLKNFIRHG~KQAR--------------------------LVTPHAEPTTNVSP-
          	 :: .. ..    . ..:                             :*   *  .  . 

MG08547.1 	--AHQPAPAAYSVQPGNDATNRAAQAGNVAAHHAEADQGVQHGIPKSKSKRIDESNIAKL
NCU09212.1	RNHQAPVQDAYSN-TQVDGHNRVAQAGHAAAHYAEADQNLAHKT-KSKTKVPDE-NIVRL
FG00786.1 	--RKTEQQQAPAVQHKNASDPSTYARSQEPAGHDYGDD--EYSRGKSKKRVDDE-KLAKL
AN4483.1  	--VHAEQQRQPQGQFAPAAGNLDAIDSKLGGGQPHSTQ---RESPEIPVKRAREAEIEQI
          	   :         .    .       .:  .    . :       :   :   *::: ::

MG08547.1 	VAEEAASKSKFPKYPGLEKWELLEKMGDGAFSNVYRARDLEGKMPGEVAIKVVRKFEMSS
NCU09212.1	VAEENASKSKFPRYPGLERWELVEKMGDGAFSNVYRARDLEG-NAGEVAIKVVRKFEMNN
FG00786.1 	IAEENASKSKFPRYPGLERWELVDKMGDGAFSNVYRARDTTG-VQGEVAIKVVRKYEMNS
AN4483.1  	IAEEKTNRTKMPHYPGLERYVLLEKMGDGAFSNVYRAKDASG-EYGEVAIKVVRKFEMNS
          	:*** :.::*:*:*****:: *::*************:*  *   **********:**..

MG08547.1 	MQ0-----------------~RANILKEVQIMRQLDHPNIIKMVDFSESKQYYYIILELA
NCU09212.1	MQ0-----------------~RANILKEVQIMRQLDHPNIIKLVDFAESRQYYYIILELA
FG00786.1 	MQ0-----------------~RSNILKEVQIMRQLDHPNIIKLVEFSESRQYYYIVLELA
AN4483.1  	TQ0SDAHLHPDFKRKPKAAE0RANILKEVQIMRQIDHPNIVKLIQFSESRQYYYIVLELC
          	 * :.:   .. . ...::. *:***********:*****:*:::*:**:*****:***.

MG08547.1 	PGGELFHQIVRLTYFSEELSRHVIVQVAKALEYLHEERGVVHR2DIKPENILFSPIPVVP
NCU09212.1	PGGELFHQIVRLTYFSEDLSRHVITQVAKALEYLHEEKGVVHR2DIKPENILFSPIPFIP
FG00786.1 	PGGELFHQIVRLTYFSEELSRHVILQVAQALEYLHEEKGVVHR2DIKPENILFEPIPMVP
AN4483.1  	PGGELFHQIVRLTYFSEDLSRHVIVQVAKAIEYLHETSGVVHR2DIKPENLLFYPTEYVP
          	*****************:****** ***:*:*****  ***** ******:** *   :*

MG08547.1 	SKQPKPKQPGDEDKVDEGEFIPGVGGGGIGQIKIADFGLSKIVWDNQTMTPCGTVGYTAP
NCU09212.1	SKHPKPKQPGDEDKVDEGEFIKGVGSGGIGQIKIADFGLSKIVWDKQTMTPCGTVGYTAP
FG00786.1 	SKHPKPKQPGDEDKVDEGEFIPGQGAGGIGRIKIADFGLSKIVWDNQTMTPCGTVGYTAP
AN4483.1  	SKHPKPRQPGDEDKVDEGEFIPGKGAGGIGVIKIADFGLSKVIWDTQTMTPCGTVGYTAP
          	**:***:************** * *.**** **********::**.**************

MG08547.1 	EIVKDERYSKSVDMWALGCVLYTLLCGFPPFYDESIEILTEKVAKGQYTFLSPWWDDISK
NCU09212.1	EIVKDERYSKAVDMWALGCVLYTLLCGFPPFYDESIEVLTEKVAKGQYTFLSPWWDDISK
FG00786.1 	EIVKDERYSKSVDMWALGCVLYTLLCGFPPFYDESIEVLTEKVAKGQYTFLSPWWDEISK
AN4483.1  	EIVKDERYSKSVDMWALGCVLYTLLCGFPPFYDESIQVLTEKVARGQYTFLSPWWDDISK
          	**********:*************************::******:***********:***

MG08547.1 	SAQDLISHLLTVDPEKRFTITEFLAHPWIRGSG----------------PTPRDEKKFAP
NCU09212.1	SAQDLISHLLCVDPEKRYTITEFLAHPWIAGSG----------------PTPRDERK-NP
FG00786.1 	SAQDLISHLLTVDPEKRYTITEFLAHPWISGNG----------------PTPREEMK-KA
AN4483.1  	SAKDLISHLLTVDPDERYTIKQFLAHPWIRQSGEETQAASDAPPLTTPAPEARNQALDAI
          	**:******* ***::*:**.:*******  .*..:.:::.:.. ::.:* .*::     

MG08547.1 	DGMLRAFDAG--RLQDGDRRYDFRSPGAVNLREVFDVGYAVHRQEEEGKRRKQ-VGTKAG
NCU09212.1	DGVLRAFDAS--KIVDGDRRYDFRSPGAVNLREVFDVGYAVHRQEEEGKRRHN-LGAKAG
FG00786.1 	DGMLRAFDAT--KFEEAGRRYDFRSPGAVNLREVFDVGYAVHRQEEEGKRRAQ-IGPKG-
AN4483.1  	AAELAPYPPASAQLSAGERPMDFRSPGAINLREVFDVGYAVHRQEEEGKRRKAGRGFRQS
          	 . * .: . ::::  . *  *******:**********************  . * : .

MG08547.1 	GAPAI-LNNLNEEDEDASMRDADSDESGEYSHKKAGANTTQSLEQSMRNANIRGEKEAQ-
NCU09212.1	VSRLVGLDENSDEGEDVAMHDVVN-----------GKQYHTSLEQSMRNAQIRDQQQEQ-
FG00786.1 	-TPARFLGGLNEEDEDEDHMEIDGQENDD----AKPNAATKALEQSMRKANIRDQEQQQQ
AN4483.1  	NPTAAFQSALNPLDEDYDDEDGIAYQTID----EEGHPPAKMQKSTQQSGDVAAMEAKM-
          	 .     .  .  .**    :    .. .              :.: :..::   :    

MG08547.1 	--RGRERESNKGAPTAVGEPQQRGYGQHSAAVTAAARQQVRDRNRQKGAFELNLDGATLL
NCU09212.1	--RGRDRERKAHPPPAAAE--QRGYGQHSAAVTAAARQQVRERNRQKGAFELNLDGATLL
FG00786.1 	QSRGRERERT-----------EKGYGQHSAAVAAAARQQVRDRNRQRGAFELNLDNATLL
AN4483.1  	--RQTNLGATSHAAQMRQSHQPRGYGQHSAQVAAAAKQNMVRNAR--APFELSLDNASLL
          	  *  :   .  ..    . . :******* *:***:*::  . *  ..***.**.*:**

MG08547.1 	GRRGVKQAPP-VA~-------~--------------------~-----------------
NCU09212.1	GRRGVKPSAARVA~-------~--------------------~-----------------
FG00786.1 	GKRNKKVPVMGV-~-------~--------------------~-----------------
AN4483.1  	EKRGRRHQEQPAI2PFATLLI2GNQTNRPRLLLQAASSRIQV1HLLLVSVQKQLVLLQSI
          	 :*. :     .  . ::    ...:. .    .::::  .       : ...    .: 

MG08547.1 	---------------
NCU09212.1	---------------
FG00786.1 	---------------
AN4483.1  	LLNRDNSDVPHSASI
          	  . ..:. . :::
```
